# Relationship between Tei index of myocardial performance and left ventricular geometry in Nigerians with systemic hypertension

**DOI:** 10.5830/CVJA-2010-050

**Published:** 2011-06

**Authors:** Adeseye A Akintunde, Patience O Akinwusi, George O Opadijo

**Affiliations:** Division of Cardiology, Ladoke Akintola University of Technology Teaching Hospital, Osogbo, Nigeria; Cardiology Clinic, Eberhards Karls University, Tubingen, Germany; Division of Cardiology, Ladoke Akintola University of Technology Teaching Hospital, Osogbo, Nigeria; Division of Cardiology, Ladoke Akintola University of Technology Teaching Hospital, Osogbo, Nigeria

**Keywords:** Tei index, hypertension, left ventricle geometry, echocardiography

## Abstract

**Introduction:**

Left ventricular geometry is associated with cardiovascular events and prognosis. The Tei index of myocardial performance is a combined index of systolic and diastolic dysfunction and has been shown to be a predictor of cardiovascular outcome in heart diseases. The relationship between the Tei index and left ventricular geometry has not been well studied. This study examined the association between the Tei index and left ventricular geometry among hypertensive Nigerian subjects.

**Methods:**

We performed echocardiography on 164 hypertensives and 64 control subjects. They were grouped into four geometric patterns based on left ventricular mass and relative wall thickness. The Tei index was obtained from the summation of the isovolumic relaxation time and the isovolumic contraction time, divided by the ejection time. Statistical analysis was done using SPSS 16.0.

**Results:**

Among the hypertensive subjects, 68 (41.4%) had concentric hypertrophy, 43 (26.2%) had concentric remodelling, 24 (14.6%) had eccentric hypertrophy, and 29 (17.7%) had normal geometry. The Tei index was significantly higher among the hypertensives with concentric hypertrophy (CH), concentric remodelling (CR) and eccentric hypertrophy (EH) compared to the hypertensives with normal geometry (0.83 ± 1.0, 0.71 ± 0.2, 0.80 ± 0.2 vs 0.61 ± 0.2, respectively). The Tei index was higher among hypertensives with CH and EH than those with CR. Stepwise regression analysis showed that the Tei index was related to ejection fraction, fractional shortening and mitral E/A ratio.

**Conclusion:**

Among Nigerian hypertensives, LV systolic and diastolic functions (using the Tei index) were impaired in all subgroups of hypertensive patients according to their left ventricle geometry compared to the control group. This impairment was more advanced in patients with concentric and eccentric hypertrophy.

## Abstract

Hypertension remains the commonest cardiovascular risk factor in Nigeria.^1^ Left ventricular adaptations to hypertension include the development of left ventricular hypertrophy and several geometric alterations.^2^,^3^ These geometric variations are associated with significant morbidity and mortality, with various studies suggesting that subjects with concentric hypertrophy have a higher incidence of cardiovascular events, and those with eccentric hypertrophy are much more at risk of progressing to heart failure than those with other left ventricular geometric patterns.^4^-^6^ Other studies suggest that major cardiovascular events are similar between the four groups of geometric patterns.^7^,^8^ However, left ventricular function has been shown to vary between the different left ventricular geometric patterns.8-10

Hypertension can lead to both systolic and diastolic abnormalities. There are several conventional indices for evaluating systolic function, such as ejection fraction, cardiac output, cardiac index and fractional shortening, while diastolic index can be assessed among others by transmitral Doppler wave pattern, isovolumic relaxation time, tissue Doppler and deceleration time. These indices are specific for systolic or diastolic function and each is associated with significant limitations with regard to their interpretation/relevance to clinical status and haemodynamic state.

The myocardial performance index, otherwise called the Tei index, is a combined index of systolic and diastolic dysfunction and has been shown to correlate significantly with other conventional indices.^11^,^12^ Whether there is any relationship between the Tei index and left ventricular geometry among blacks is not well known. Hence this study aimed to describe the relationship between left ventricular geometry and the myocardial performance index among hypertensive Nigerian subjects.

## Methods

Patients and controls were consecutively recruited from the echocardiographic laboratory of our hospital. This study was cross-sectional and comprised 164 hypertensive subjects seen at the cardiology clinic of our hospital. They had had complete echocardiography done on them, including two-dimensional, Doppler and M-mode echocardiography. Adult hypertensive subjects who were at least 18 years old and who were willing to participate in the study were included.

Blood pressure was measured in the clinic with an Accosson’s sphygmomanometer and hypertension was diagnosed when blood pressure was persistently ≥ 140 and/or ≥ 90 mmHg and/or the use of antihypertensive therapy according to standardised procedures and guidelines.^13^ Systolic and diastolic blood pressure was taken as the first and fifth korotkoff sounds, respectively. Other clinical information obtained included weight (in kilograms), height (in metres), body mass index, age and gender.

Sixty-four normotensives were used as controls. They were randomly recruited from patients’ relatives and hospital staff who were willing to participate. They were well matched in age and gender distribution with the hypertensive subjects. Patients with diabetes mellitus or a history of heart or renal failure were excluded from the study. Determinations of the Tei indices were done differently without access to the clinical data of the subjects.

## Echocardiography

All the subjects had transthoracic echocardiography done according to the recommendation of the American Society of Echocardiography.^14^ Patients were placed in the left lateral decubitus position. Left ventricular measurements such as wall and chamber dimensions were obtained in systole and diastole. They included left ventricular internal dimension in diastole (LVIDd) and systole (LVIDs), posterior wall thickness in diastole (PWTd), and interventricular septal thickness in diastole (IVSd). Left ventricular mass (LVM) was calculated from the measurements of the left ventricle (LV) using the equation:

LVM (g) = 0.81 [1.04 (interventricular septal thickness + posterior wall thickness + LV end-diastolic internal dimension)3 – (LV end-diastolic internal dimension)3] + 0.6.^15^

LVM index (LVMI) was calculated as LVM/height (m)^2.7^. Correcting LVM for height ^2.7^ has been shown to minimise the effect of gender, race, age and obesity on the validity of various parameters for the diagnosis of left ventricular hypertrophy (LVH), for which many parameters exist.^16^ LVH was defined as LVMI > 51 g/m^2.7^.

LV geometry was determined after calculation of the relative wall thickness (RWT) using the formula:

$\frac{2\;\times \;posterior\;wall\;thickness\;}{LV\;end-diastolic\;internal\;dimension.\;}$17

RWT was considered abnormal if it was ≥ 0.45.17

Four left ventricular geometric patterns were described based on RWT and left ventricular mass index (LVMI): normal geometry, concentric remodelling, eccentric hypertrophy and concentric hypertrophy. LV geometry was defined as concentric hypertrophy (elevated LVMI and RWT), concentric remodelling (normal LVMI and elevated RWT), eccentric hypertrophy (increased LVMI and normal RWT) and normal geometry (normal LVMI and RWT).

The Tei index reflects both systolic and diastolic function. It was defined as the sum of the isovolumic relaxation time and isovolumic contraction time divided by the ejection time obtained from the left ventricular inflow and outflow.^11^ The isovolumic relaxation time was determined from the apical five-chamber view as the time from the end of left ventricular ejection to the beginning of the early mitral inflow (E) wave. Isovolumic contraction time was defined as the time from the peak of the R wave or the end of the late atrial filling (A) wave to the beginning of left ventricular ejection.

## Statistical analysis

The Statistical Package for Social Sciences, SPSS 16.0 (Chicago Ill.) was used for the study. Quantitative data were summarised using means ± standard deviation (SD) while qualitative data were summarised using percentages and proportions. The Student’s *t*-test and chi-squares were used as appropriate for intergroup comparisons. Correlation analysis was used for univariate associations between numerical variables. Values with *p* < 0.05 were taken as statistically significant. Ethical approval was obtained for the study.

## Results

The clinical characteristics of the study population are shown in Table 1. The hypertensives and the control population were well matched in age and gender distribution. Left ventricular wall thickness, isovolumic relaxation and contraction time, body mass index, left ventricular mass index, relative wall thickness and Tei index were significantly higher among the hypertensive subjects than the controls. Left ventricular internal diastolic and systolic dimensions and ejection times were however similar between the hypertensive and control groups.

**Table 1.** Clinical And Echocardiographic Parameters Of Study Participants

*Variable*	*Hypertensives (164)*	*Controls (64)*	p
Age (years)	56.6 ± 12.5	55.2 ± 7.8	0.408
Gender F (%)	78 (47.6%)	31 (48.3%)	0.675
SBP (mmHg)	147.9 ± 24.0	125.1 ± 15.1	0.000**
DBP (mmHg)	89.9 ± 11.7	78.9 ± 12.1	0.000**
LVIDd (mm)	45.9 ± 9.0	45.3 ± 8.8	0.660
LVIDs (mm)	31.0 ± 11.1	32.4 ± 8.0	0.389
IVSd (mm)	14.5 ± 4.6	10.3 ± 3.6	0.000**
PWTd (mm)	12.3 ± 5.9	10.3 ± 2.7	0.022*
ET (msec)	275.0 ± 56.8	284.4 ± 36.1	0.239
IVCT (msec)	101.1 ± 46.6	88.0 ± 25.6	0.012*
IVRT (msec)	99.4 ± 28.6	79.9 ± 14.2	0.000**
BMI (kg/m^2^)	26.1 ± 5.3	23.3 ± 4.1	0.000**
LVMI (g/m^2.7^)	106.5 ± 55.3	42.3 ± 19.8	0.038**
RWT	0.56 ± 0.5	0.42 ± 0.1	0.025*
Tei index	0.77 ± 0.68	0.40 ± 0.07	0.000**

*Statistically significant. SBP: systolic blood pressure, DBP: diastolic blood pressure, LVIDd: left ventricular internal dimension in diastole, LVIDs: left ventricular internal dimension in systole, PWTd: posterior wall thickness in diastole, ET: ejection time, IVCT: isovolumic contraction time, IVRT: isovolumic relaxation time, BMI: body mass index, LVMI: left ventricular mass index, RWT: relative wall thickness, F: female.

Table 2 shows the mean age and echocardiographic parameters among the four left ventricular geometric patterns. Concentric hypertrophy was the commonest pattern of abnormal geometry (41.4%), followed by concentric remodelling (26.2%), and eccentric hypertrophy was demonstrated in 14.6% of the hypertensive population. Only 17.7% of the hypertensive population had normal geometry. Hypertensives with concentric hypertrophy were likely to be older. The mean Tei index was significantly higher in patients with each abnormal geometric pattern, compared to the hypertensives with normal geometry. Those with concentric hypertrophy had the highest Tei index and were significantly different from those with concentric remodelling. However, there was no significant difference in the Tei index between those with eccentric and concentric hypertrophy.

**Table 2.** Clinical And Echocardiographic Parameters Among Various Left Ventricular Geometric Patterns In Hypertensives

*Variable*	*Normal geometry (29)*	*Concentric remodelling (43)*	*Concentric hypertrophy (68)*	*Eccentric hypertrophy (24)*
Age (years)	54.4 ± 12.0	55.1 ± 12.2	58.3 ± 12.1	56.1 ± 11.0
IVSd (mm)	10.7 ± 3.0	12.3 ± 2.5^‡^	15.7 ± 7.9^‡†^	13.7 ± 6.1^‡^
PWTd (mm)	10.4 ± 2.3	11.5 ± 1.4^‡^	15.0 ± 1.9^‡†^	10.4 ± 2.2^‡^
ET (msec)	283.0 ± 32.8	275.1 ± 37.8	280.0 ± 59.5	243.0 ± 86.7^†^
IVRT (msec)	97.1 ± 25.6	97.8 ± 25.9	100.7 ± 31.9	101.9 ± 30.1
IVCT (msec)	99.1 ± 35.4	94.8 ± 39.3	103.3 ± 56.1^†^	105.0 ± 37.9
RWT	0.38 ± 0.05	0.60 ± 0.11^‡^	0.70 ± 0.69^‡†^	0.36 ± 0.07^†^
Tei index	0.61 ± 0.2	0.71 ± 0.2^‡^	0.83 ± 1.0^‡†^	0.80 ± 0.2^‡^

^‡^*p* < 0.05 vs normals; ^†^*p* < 0.05 vs concentric remodelling. PWTd: posterior wall thickness in diastole, ET: ejection time, IVCT: isovolumic contraction time, IVRT: isovolumic relaxation time, RWT: relative wall thickness.

Univariate correlation coefficients of the echocardiographic parameters with Tei index (myocardial performance index) are shown in Table 3. Ejection fraction, fractional shortening, mitral E/A ratio and left ventricular internal systolic dimensions were significantly correlated with the Tei index. Multiple regression analysis revealed that the Tei index was significantly related to only ejection fraction (–0.232), fractional shortening (*r* = –0.142) and mitral E/A ratio (*r* = –0.280) (Table 4).

**Table 3.** Correlation Of Echocardiographic Parameters With Tei Index

*Variable*	*Correlation*	p
LVIDd (mm)	0.124	0.114
LVIDs (mm)	0.159	0.044*
EF	–0.209	0.008*
Mitral E/A ratio	–0.198	0.006*
FS	–0.187	0.018*
IVSd	–0.013	0.822
PWTd	0.018	0.818
RWT	–0.018	0.816

*Statistically significant. SBP: systolic blood pressure, DBP: diastolic blood pressure, LVIDd: left ventricular internal dimension in diastole, LVIDs: left ventricular internal dimension in systole, PWTd: posterior wall thickness in diastole, ET: ejection time, IVCT: isovolumic contraction time, IVRT: isovolumic relaxation time, BMI: body mass index, LVMI: left ventricular mass index, RWT: relative wall thickness.

**Table 4.** Correlation Coefficients Of Linear Regression Analysis Between The Tei Index And Other Parameters

*Clinical parameter*	*Correlation coefficient*	p
Age	0.101	0.218
SBP	0.101	0.220
DBP	0.049	0.551
BMI	0.145	0.079
PWTd	0.018	0.830
IVSd	–0.005	0.954
MEARAT	–0.280	0.045*
EF	–0.232	0.004*
FS	–0.142	0.032*
RWT	0.001	0.886

*Statistically significant. SBP: systolic blood pressure, DBP: diastolic blood pressure, LVIDd: left ventricular internal dimension in diastole, LVIDs: left ventricular internal dimension in systole, PWTd: posterior wall thickness in diastole, ET: ejection time, IVCT: isovolumic contraction time, IVRT: isovolumic relaxation time, BMI: body mass index, LVMI: left ventricular mass index, RWT: relative wall thickness.

## Discussion

This study revealed some important findings. Firstly, the Tei index was significantly different between hypertensive subjects with normal and those with abnormal left ventricular geometry. Secondly, concentric hypertrophy was the commonest abnormal pattern of left ventricular geometric pattern in this study, followed by concentric remodelling and then eccentric hypertrophy. Only 17.7% of the hypertensive subjects had normal left ventricular geometry. Thirdly, the Tei index was highest among hypertensive subjects with concentric hypertrophy, closely followed by those with eccentric hypertrophy, with no significant difference between the two groups. Fourthly, multiple regression analysis suggests that there was no significant association between left ventricular geometric pattern and Tei index.

The pattern of Tei index in this study therefore indicated the pattern/severity of combined systolic and diastolic function associated with each type of left ventricular geometry among these hypertensive subjects and was not a function of the left ventricular geometry itself. Earlier studies have found concentric and eccentric hypertrophy to be associated with a higher degree of cardiovascular events, and systolic and diastolic dysfunction.^4^-^6^ The Tei index was also not associated with age, left ventricular mass or body mass index in this study population.

Ejection fraction, fractional shortening and mitral E/A ratio were the main echocardiographic parameters associated with Tei index among hypertensive Nigerian subjects. This study therefore demonstrates the potential clinical usefulness of the Tei index in stratifying left ventricular systolic and diastolic dysfunction across all age strata and patient groups, notwithstanding the body mass index and left ventricular mass.

Use of the Tei index for assessing cardiac performance has a potential clinical advantage over the use of other classical echocardiographic indices for estimating left ventricular diastolic and systolic function. Ejection fraction and left ventricular volumes, which are measures of systolic function, are prone to large errors when the ellipsoid shape of the heart is altered. Age, rhythm, preload and afterload changes affect the transmitral Doppler signal, which is a measure of diastolic function. Therefore the use of these conventional indices when left ventricular geometry is altered may be further error-prone.

Left ventricular geometric pattern has been shown to be related to diastolic and systolic function.^8^-^10^ However, the Tei index is not affected by these factors and is therefore more likely to be a better estimate of systolic and diastolic function in a large group of the population, irrespective of confounding factors, as shown in various studies.^11^,^18^,^19^ This study further corroborates other studies that have shown that the Tei index is independent of flow haemodynamics, preload, afterload, age and left ventricular geometry among a variety of subjects, including paediatric subjects.^11^,^19^-^21^

Several geometric variations are cardiac adaptations to pressure and volume changes in hypertension, with the aim of maintaining normal function. The early phases of altered geometry are associated with compensated left ventricular function to a near-normal level. This study revealed that the Tei index was significantly higher among hypertensive subjects with concentric hypertrophy compared to those with concentric remodelling. Concentric hypertrophy has been associated with an increase in cardiovascular events associated with systolic and diastolic dysfunction in various studies.^8^,^9^,^12^ The Tei index was similar between hypertensive subjects with concentric hypertrophy and those with eccentric hypertrophy. In some studies, concentric and eccentric hypertrophy have been shown to be more associated with impaired diastolic function than with concentric remodelling.^22^,^23^

Other studies have reported the relationship between left ventricular geometry and systolic function in other populations. 4,8,12 Yilmaz *et al*. reported in 2004 that the systolic and diastolic LV functions (using Tei index of myocardial performance) were impaired in all subgroups of hypertensive patients according to their left ventricle geometry compared to the control group. This impairment was more advanced in patients with concentric hypertrophy than in those with other LV geometric patterns.^24^ Therefore, it can be concluded that abnormal left ventricular geometric adaptation to hypertension is associated with systolic and diastolic dysfunctions, which are reflected accurately by the Tei index of myocardial performance among hypertensive Nigerian subjects.

In terms of the limitations of this study, it does not provide any information on the prognostic potential of the Tei index among hypertensive subjects. Therefore, a prospective study to determine the prognostic potential of the Tei index among black hypertensive subjects would be relevant. Also, the impact of antihypertensive therapy on the Tei index was not studied. It has been shown that the Tei index is able to detect improvement in systolic function in heart failure patients using carvedilol, and improvement in systolic function in subjects with acute myocardial infarction.^25^-^27^
